# Using molecular network analysis to understand current HIV-1 transmission characteristics in an inland area of Yunnan, China

**DOI:** 10.1017/S0950268823001140

**Published:** 2023-07-18

**Authors:** Rui Cao, Shouxiong Lei, Huichao Chen, Yanling Ma, Jie Dai, Lijuan Dong, Xiaomei Jin, Min Yang, Pengyan Sun, Yawen Wang, Yuying Zhang, Manhong Jia, Min Chen

**Affiliations:** 1 School of Public Health, Kunming Medical University, Kunming, China; 2 Division for AIDS/STD Control and Prevention, Zhaotong Center for Disease Control and Prevention, Zhaotong, China; 3 Institute for AIDS/STD Control and Prevention, Yunnan Center for Disease Control and Prevention, Kunming, China; 4 Health Laboratory Center, Yunnan Center for Disease Control and Prevention, Kunming, China

**Keywords:** assortativity, HIV-1, molecular network, spatial analysis

## Abstract

HIV-1 molecular surveillance provides a new approach to explore transmission risks and targeted interventions. From January to June 2021, 663 newly reported HIV-1 cases were recruited in Zhaotong City, Yunnan Province, China. The distribution characteristics of HIV-1 subtypes and HIV-1 molecular network were analysed. Of 542 successfully subtyped samples, 12 HIV-1 strains were identified. The main strains were CRF08_BC (47.0%, 255/542), CRF01_AE (17.0%, 92/542), CRF07_BC (17.0%, 92/542), URFs (8.7%, 47/542), and CRF85_BC (6.5%, 35/542). CRF08_BC was commonly detected among Zhaotong natives, illiterates, and non-farmers and was mostly detected in Zhaoyang County. CRF01_AE was frequently detected among married and homosexual individuals and mostly detected in Weixin and Zhenxiong counties. Among the 516 *pol* sequences, 187 (36.2%) were clustered. Zhaotong natives, individuals aged ≥60 years, and illiterate individuals were more likely to be found in the network. Assortativity analysis showed that individuals were more likely to be genetically associated when stratified by age, education level, occupation, and reporting area. The genetic diversity of HIV-1 reflects the complexity of local HIV epidemics. Molecular network analyses revealed the subpopulations to focus on and the characteristics of the risk networks. The results will help optimise local prevention and control strategies.

## Introduction

Since the recognition of acquired immune deficiency syndrome (AIDS) in 1981 and the discovery of human immunodeficiency virus (HIV) in 1983, the HIV epidemic has remained a major public health problem worldwide [1]. According to the UNAIDS data [2], by the end of 2021, 38.4 million people were living with HIV globally. In 2021, 1.5 million people were newly infected with HIV and 650,000 people died from AIDS-related illnesses. Meanwhile, the rapid replication rate, error-prone reverse transcription, and frequent genetic recombination make HIV highly variable [3, 4], hindering the development of biomedical approaches for prevention and treatment [5, 6].

The transmission of HIV/AIDS is complex and influenced by a variety of social and biological factors. Social discrimination and privacy issues make it difficult to trace the sources of infection and effectively stop transmission. Therefore, the accurate identification of HIV transmission is the first step in public health interventions [7]. Even with the 90–90–90 strategy for HIV/AIDS control and prevention, the key measures are to identify populations at high risk of HIV transmission and to prioritise resources for treatment and prevention [8]. In recent years, the use of HIV-1 molecular network analysis has increased [9–12]. Phylogenetic analysis, genetic distance analysis, and a combination of both methods are used to identify HIV-1 molecular networks [9, 13]. In the phylogenetic tree approach, a cluster is identified as a molecular network if its node support value is within a specified range. The gene distance method uses a specific gene distance threshold between sequences to determine the molecular network [14]. As the genetic distance is related to the evolution of the virus, it can reflect the proximity of the time of transmission [14]. Thus, some HIV-1 molecular network studies using phylogenies have also added a genetic distance component. In practice, the choice of methods depends on the characteristics of the data and research objectives. Transmission networks can be further inferred from molecular networks when combined with epidemiological information, providing a basis for risk assessment [15–18]. In addition to retrospective analysis, real-time molecular surveillance provides a tool for identifying and responding to rapid HIV transmission in communities, which can slow or even prevent HIV outbreaks [12]. Consequently, real-time detection and response to HIV clusters are considered a key strategy for ending the HIV epidemic [11].

Yunnan Province is located in southwest China bordering Myanmar, Laos, and Vietnam. Because of its proximity to the Golden Triangle, HIV entered Yunnan through drug traffic. In 1989, HIV epidemics were first reported among intravenous drug users in the western border area of Yunnan [19]. After that, the HIV epidemic developed rapidly, and Yunnan became one of the areas hardest hit by HIV/AIDS in China. After 2006, heterosexual contact replaced intravenous drug use as the predominant mode of transmission in Yunnan [20]. According to a series of retrospective molecular epidemiological studies, the major HIV-1 strains circulating in China are associated with Yunnan Province [19, 21–23]. For example, CRF07_BC and CRF08_BC were generated by the recombination of subtypes B and C in Yunnan and were transmitted to other parts of China [22]. In addition, Yunnan has become a genetic reservoir for HIV-1, and to date, more than 17 circulating recombinant forms (CRFs) have been identified in Yunnan.

Although the HIV-1 epidemic started in border areas, it gradually spread inland. Zhaotong City, which covers 11 counties with a population of 501.4 million, is located in the northeastern part of Yunnan Province, bordering Sichuan Province to the northwest and Guizhou Province to the east and is an important gateway to Sichuan and Guizhou provinces. By the end of 2020, the cumulative number of people living with HIV/AIDS (PLWHA) in Zhaotong City was 7,805, of which 7,188 (92.1%) were receiving antiretroviral therapy (ART). In 2020, the number of newly reported infections in Zhaotong City ranked third in Yunnan Province. Meanwhile, heterosexual transmission has continued to increase. In particular, more than 30% of cases were detected late, with CD4^+^T lymphocyte counts below 200 cells/μl. This suggests that the local HIV epidemic is still complex and that the risk of transmission remains. To control the HIV-1 epidemic, it is necessary to understand its transmission characteristics and risk networks. In combination with the 90–90–90 strategy, effective detection and intervention measures should be developed. To this end, a molecular epidemiological study combined with social network analysis was conducted in Zhaotong City.

## Methods

### Study participants and sample collection

From January to June 2021, 671 HIV/AIDS cases were newly reported in Zhaotong City, of which 663 (98.8%) were recruited for this study. The inclusion criteria were as follows: (1) AIDS patients aged 15 years and above and (2) those willing to participate in the survey and sign an informed consent form. This study was approved by the Research Ethics Review Committee of the Yunnan Center for Disease Control and Prevention (YNCDC-ER-202108). Written informed consent was provided by adults and legal guardians. All methods were performed in accordance with relevant guidelines and regulations.

### Amplification and sequencing of HIV-1 gene fragments

HIV-1 viral RNA was extracted from 140 μl of plasma using a QIAamp Viral RNA Kit (Qiagen, Germany). The *gag* (HXB2:781–1861), *pol* (HXB2:2147–3462), and *env* (HXB2:7002–7541) genes were amplified by nested polymerase chain reaction (PCR). The first round of PCR amplification was performed using the One Step RNA PCR Kit (Takara, Dalian, China), and the second round of amplification was performed using 2 × Taq PCR MasterMix (Tiangen, Beijing, China). The primers and procedures used for nested PCR were described previously [24]. Amplified products were sent to SinoGenoMax Co. (Beijing, China) for Sanger sequencing with ABI 3730XL.

### Sequence analysis

Sequences were assembled using Sequencher 5.1 (Gene Codes, Ann Arbor, MI). The BioEdit software (version 7.0) was used for sequence alignment and cleaning. Neighbour-joining phylogenetic trees were constructed using the Kimura 2-parameter model with 1000 bootstrap replicates in MEGA-X. Subtype reference alignments were downloaded from HIV databases (www.hiv.lanl.gov), which includes four genomes for each subtype and CRF. Sequences with unclear subtyping were further analysed using the REGA HIV subtyping tool (http://dbpartners.stanford.edu:8080/RegaSubtyping/stanford-hiv/typingtool/) and the Recombination Identification Program (RIP, https://www.hiv.lanl.gov/content/sequence/RIP/RIP.html). The final subtyping result for each sample was based on at least any two segments of *gag*, *pol*, and *env* genes (Supplementary Table S1).

### Spatial analysis

The distributions of major HIV-1 strains are presented using dot density maps. The percentage of a given HIV strain is shown as the number of dots, with one dot representing 0.03% of the total number of subtyped cases. Global Moran’s *I* was calculated to determine the global spatial autocorrelation of HIV-1 strains, which varies between −1 and 1. Moran’s *I* > 0 indicates spatial clustering, and Moran’s *I* < 0 indicates spatial dispersal. If the global Moran’s *I* was significant, the local Moran’s *I* was calculated. Local Moran’s *I* > 0 and *Z* ≥ 1.96 indicate positive spatial correlations (high–high and low–low clusters). Local Moran’s *I* < 0 and *Z* ≤ −1.96 indicate negative spatial correlations (high–low and low–high clusters). Data on administrative boundaries were downloaded from the National Catalogue Service for Geographic Information (https://www.webmap.cn; accessed on November 11, 2022). Spatial analysis was performed using GeoDa 1.20 and QGIS 3.28.

### Construction and analysis of HIV-1 molecular network

HIV-1 molecular clusters were investigated using phylogenetic analysis combined with genetic distance analysis. A maximum likelihood (ML) phylogenetic tree was constructed with 516 *pol* sequences using FastTree software with the generalised time-reversible model. The reliability of nodes was assessed using the Shimodaira–Hasegawa (SH) test with 1000 resamples, from which transmission clusters were extracted using Cluster Picker software [25]. To select the optimal genetic distance, the pairwise genetic distance was varied from 0.5 to 5.0%, with an interval of 0.5% when the node support threshold was set at ≥90%. As shown in Supplementary Figure S1, the growth rate of the number of clusters decreased significantly when the genetic distance was >2.5%. Therefore, a genetic distance of 2.5% was selected. The HIV-1 molecular network was visualised using the igraph R package.

The assortativity coefficient measures the degree of homophyly of a network based on a given node attribute [26]. The assortativity coefficient ranges from −1.0 to 1.0. When the assortativity coefficient is greater than zero, individuals in the same category tend to cluster. Individuals from different categories tend to cluster when the assortativity coefficient is less than 0. When the assortativity coefficient is zero, the category does not influence individual clustering. In this study, the assortativity coefficient was calculated using R package igraph. For each category, a null expectation was generated by randomly permuting trait labels across the static network 1000 times and recalculating assortativity [27].

### Sequence data

The sequences obtained in this study were submitted to NCBI GenBank under accession numbers OP979115-OP980539.

### Statistical analysis

Statistical analysis was conducted using SPSS software (version 19.0; SPSS Inc. Chicago, IL). Categorical variables were compared using the 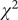 test. Factors of interest were analysed using logistic regression analysis. All tests were two-tailed, and a *p* value <0.05 was considered statistically significant.

## Results

### Demographic characteristics of the participants

From January to June 2021, 663 newly diagnosed HIV-1 cases were recruited from Zhaotong City. From the plasma samples, 495 *gag*, 516 *pol*, and 414 *env* sequences were obtained. After phylogenetic analysis (Supplementary Figures S2–S4), 542 samples were subtyped. Reasons for some sequences not successfully amplified included poor conditions for storing and transporting samples, or low viral loads. There were no significant differences in the demographic characteristics between samples with and without genotypes (Supplementary Table S2).

Of the 542 participants, 91.7% (497/542) were from Zhaotong, 1.3% (7/542) were from other cities in Yunnan Province, and 7.0% (38/542) were from other provinces. The median age was 50 (range, 17–83) years. The proportion of males and females was 72.3% (392/542) and 27.7% (150/542), respectively. In total, 91.7% (497/542) of the participants were of Han ethnicity. Those with a primary education were the most common, accounting for 60.3% (327/542). The majority of the participants were married, accounting for 52.8% (286/542). Farming was the main occupation, accounting for 83.0% (450/542) of the sample. Heterosexual transmission was the predominant mode of transmission, accounting for 98.3% (533/542) of the cases.

### HIV-1 genotypes detected in the participants

Twelve HIV-1 strains were detected in the 542 participants. CRF08_BC was the predominant strain (47.0%, 255/542), followed by CRF01_AE (17.0%, 92/542), CRF07_BC (17.0%, 92/542), URFs (8.7%, 47/542), CRF85_BC (6.4%, 35/542), CRF125_0107 (1.8%, 10/542), Subtype B (0.5%, 3/542), Subtype C (0.4%, 2/542), CRF55_01B (0.4%, 2/542), CRF64_BC (0.4%, 2/542), CRF101_01B (0.2%, 1/542), and CRF118_BC (0.2%, 1/542). URFs included BC recombinants (48.9%, 23/47), CRF01_AE/C (19.1%, 9/47), CRF08_BC/CRF07_BC (12.8%, 6/47), CRF01_AE/BC (6.4%, 3/47), CRF01_AE/CRF08_BC (4.3%, 2/47), CRF85_BC/CRF08_BC (4.3%, 2/47), CRF01_AE/ CRF07_BC (2.1%, 1/47), and CRF01_AE/B (2.1%, 1/47).

### Demographic characteristics of HIV-1 genotypes

The demographic characteristics of the main HIV-1 genotypes were analysed (Supplementary Table S3 and Table 1). Multivariate logistic analysis showed that CRF08_BC was more likely to be detected among the Zhaotong natives (odds ratio (OR) (95% confidence interval (CI)):2.801 (1.279–6.132)), illiterate (OR (95% CI):4.311 (1.463–12.703)), and non-farmers (OR (95% CI):2.603(1.496–4.528)). CRF01_AE was more likely to be detected in married persons (OR (95% CI):1.983(1.136–3.463)) and those infected through non-heterosexual contact (OR (95% CI):6.573(1.334–32.391)). The distribution of CRF07_BC, URFs, and CRF85_BC showed no significant demographic characteristics.

**Table 1.** Demographic characteristics associated with HIV-1 genotypes (multivariate analysis)

Characteristic			Total	Number of specific genotype (%)	Multivariate analysis	
					*p*	OR (95%CI)
CRF01_AE						
Age (years)					0.097	
≥60			105	19 (18.1%)	–	1.000
50–59			173	34 (19.7%)	0.844	1.066 (0.563–2.018)
40–49			149	19 (12.8%)	0.217	0.638 (0.312–1.302)
30–39			85	10 (11.8%)	0.253	0.603 (0.254–1.436)
<30			30	10 (33.3%)	0.191	2.020 (0.704–5.801)
Marital status					0.032	
Divorced/widowed			174	20 (11.5%)	–	1.000
Married			286	59 (20.6%)	0.016	1.983 (1.136–3.463)
Unmarried			82	13 (15.9%)	0.807	1.117 (0.460–2.711)
Infection routes					0.053	
Heterosexual contact			533	87 (16.3%)	–	1.000
Homosexual contact			7	4 (57.1%)	0.021	6.573 (1.334–32.391)
Others			2	1 (50.0%)	0.410	3.273 (0.195–55.033)
CRF07_BC						
Registered residence					0.079	
Zhaotong			497	78 (15.7%)	–	1.000
Other cities in Yunnan			7	1 (14.3%)	0.978	1.031 (0.117–9.058)
Other provinces			38	13 (34.2%)	0.024	2.360 (1.118–4.982)
Age (years)					0.134	
≥60			105	11 (10.5%)	–	1.000
50–59			173	22 (12.7%)	0.638	1.207 (0.551–2.646)
40–49			149	25 (16.8%)	0.337	1.471 (0.669–3.237)
30–39			85	23 (27.1%)	0.032	2.504 (1.082–5.795)
<30			30	11 (36.7%)	0.070	2.883 (0.917–9.063)
Marital status					0.081	
Married			286	36 (12.6%)	–	1.000
Divorced/widowed			174	31 (17.8%)	0.091	1.593 (0.929–2.733)
Unmarried			82	25 (30.5%)	0.050	1.932 (0.999–3.738)
Education					0.163	
Illiteracy			100	8 (8.0%)	–	1.000
Primary school			327	59 (18.0%)	0.055	2.178 (0.982–4.832)
Junior middle school			90	17 (18.9%)	0.227	1.796 (0.695–4.645)
Senior middle school and above			25	8 (32.0%)	0.047	3.406 (1.015–11.426)
CRF08_BC						
Registered residence					0.036	
Other provinces			38	10 (26.3%)	–	1.000
Zhaotong			497	241 (48.5%)	0.010	2.801 (1.279–6.132)
Other cities in Yunnan			7	4 (57.1%)	0.275	2.683 (0.455–15.803)
Age (years)					0.234	
<30			30	6 (20.0%)	–	1.000
30–39			85	36 (42.4%)	0.079	2.578 (0.897–7.410)
40–49			149	74 (49.7%)	0.032	3.110 (1.104–8.763)
50–59			173	89 (51.4%)	0.047	2.885 (1.012–8.224)
≥60			105	50 (47.6%)	0.136	2.292 (0.771–6.817)
Marital status					0.346	
Unmarried			82	29 (35.4%)	–	1.000
Married			286	138 (48.3%)	0.244	1.400 (0.795–2.467)
Divorced/widowed			174	88 (50.6%)	0.146	1.568 (0.855–2.874)
Education					0.009	
Senior middle school and above			25	9 (36.0%)	–	1.000
Junior middle school			90	36 (40.0%)	0.247	1.861 (0.650–5.331)
Primary school			327	147 (45.0%)	0.132	2.183 (0.790–6.038)
Illiteracy			100	63 (63.0%)	0.008	4.311 (1.463–12.703)
Occupation						
Farming			450	202 (44.9%)	–	1.000
Others			92	53 (57.6%)	0.001	2.603 (1.496–4.528)

### Spatial autocorrelation analysis of HIV-1 genotypes

**Figure 1.**
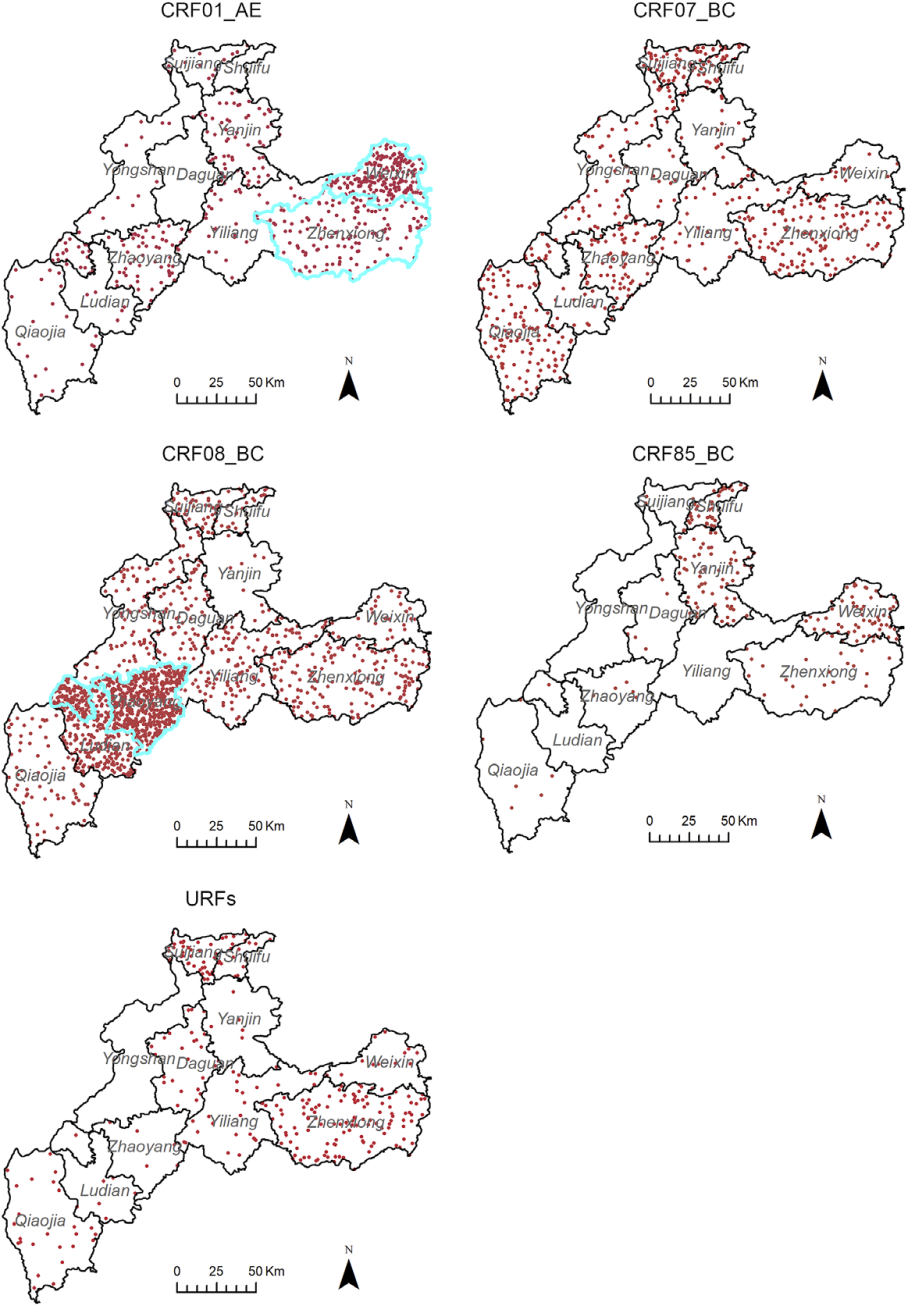
Spatial distribution of the major HIV-1 genotypes in Zhaotong City. The dot density maps for CRF01_AE, CRF07_BC, CRF08_BC, CRF85_BC, and URFs. One dot represented 0.03% of the total subtyped cases. According to the local spatial autocorrelation analysis, the areas outlined in blue are the high–high aggregation area. Administrative boundary data were downloaded from the National Catalogue Service for Geographic Information (https://www.webmap.cn). The map content approval number: ZhaotongS(2022)6.

As shown in Figure 1, CRF01_AE was mainly distributed in the eastern region, and CRF08_BC was mainly distributed in the southeastern region. CRF85_BC was mainly distributed in three counties bordering Sichuan Province in the north.

Global spatial autocorrelation analysis of key HIV-1 strains at the county level showed that CRF07_BC (Moran’s *I* = −0.507, *Z* = −1.217, *p* = 0.224), CRF85_BC (Moran’s *I* = 0.306, *Z* = 1.192, *p* = 0.233), and URFs (Moran’s *I* = −0.180, *Z* = −0.385, *p* = 0.700) tended to be randomly distributed. However, CRF01_AE (Moran’s *I* = 0.768, *Z* = 2.903, *p* = 0.004) and CRF08_BC (Moran’s *I* = 0.272, *Z* = 2.013, *p* = 0.044) showed non-random positive correlation distributions, suggesting a tendency towards spatial clustering. Local spatial autocorrelation analysis showed that the high–high cluster area of CRF01_AE was located in Weixin and Zhenxiong counties and that of CRF08_BC was located in Zhaoyang County (Figure 1).

### HIV-1 molecular network analysis

**Figure 2.**
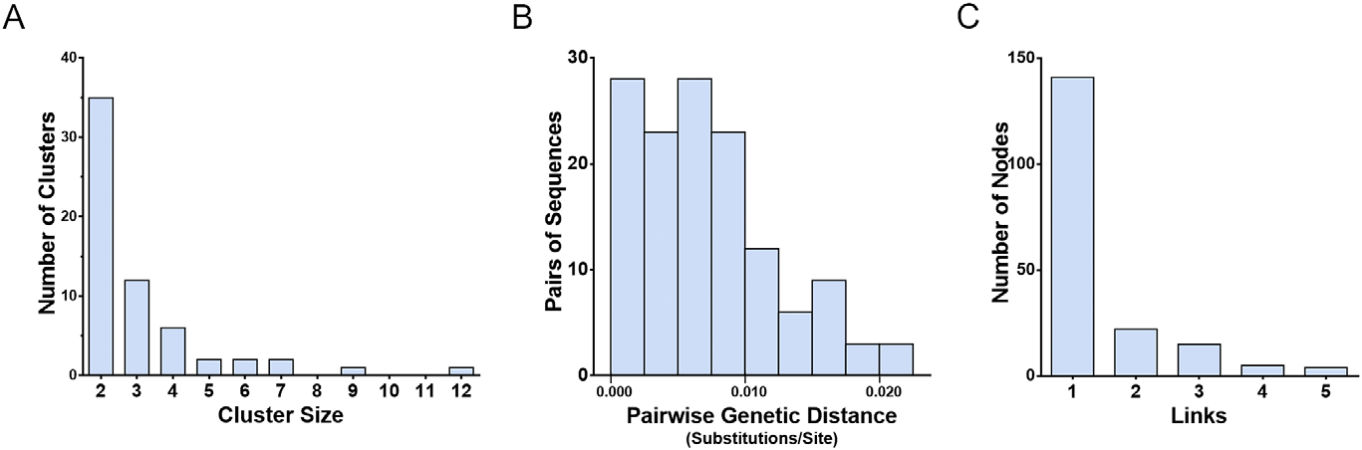
Characteristics of the HIV-1 molecular network in Zhaotong City. (a) Distribution of genetic transmission clusters by cluster size. (b) Distribution of sequence pairs by genetic distance. (c) Distribution of nodes in clusters by links.

To explore HIV-1 transmission characteristics in the local area, an HIV-1 molecular network was constructed. Among the 516 *pol* sequences, 187 (36.2%) were detected in 61 clusters, with a genetic distance threshold of 2.5%. Cluster sizes ranged from 2 to 12, with 35 (57.4%) having a cluster size of two. The median pairwise genetic distance was 0.0067 substitutions/site (interquartile range: 0.0033–0.0100 substitutions/site). Of the 187 nodes, 141 (75.4%) had one link and 46 (24.6%) had two or more links (Figure 2).

Factors associated with joining the network were analysed using logistic regression (Table 2). Multivariate analysis showed that the Zhaotong natives (OR (95% CI):2.574 (1.014–6.534)), those aged 60 years and older (OR (95% CI):6.081 (1.560–23.699)), married (OR (95% CI):2.206 (1.100–4.425)), and illiterate (OR (95% CI):4.681 (1.155–18.962)) were more likely to be found in the network.

**Table 2.** Associated demographic factors of the individuals clustering in the HIV-1 molecular network

Characteristic		Total	Within networks (%)	Univariate analysis		Multivariate analysis	
				*p*	OR(95%CI)	*p*	OR(95%CI)
Registered residence				0.030		0.138	
Other provinces		38	6 (15.8%)	–	1.000	–	1.000
Zhaotong		471	181 (38.4%)	0.008	3.329 (1.365–8.118)	0.047	2.574 (1.014–6.534)
Other cities in Yunnan		7	0 (0.0%)	0.999	–	0.999	–
Gender				0.198			
Male		376	130 (34.6%)	–	1.000		
Female		140	57 (40.7%)	0.198	1.300 (0.872–1.936)		
Age (years)				<0.001		<0.001	
<30		30	3 (10.0%)	–	1.000	–	1.000
30–39		77	14 (18.2%)	0.306	2.000 (0.531–7.531)	0.763	1.237 (0.310–4.939)
40–49		150	52 (34.7%)	0.013	4.776 (1.383–16.491)	0.123	2.809 (0.756–10.438)
50–59		161	61 (37.9%)	0.007	5.490 (1.597–18.868)	0.143	2.703 (0.716–10.210)
≥60		98	57 (58.2%)	<0.001	12.512 (3.554–44.045)	0.009	6.081 (1.560–23.699)
Race/ethnicity				0.392			
Han		476	170 (35.7%)	–	1.000		
Others		40	17 (42.5%)	0.392	1.330 (0.692–2.560)		
Marital status				0.001		0.052	
Unmarried		78	13 (16.7%)	–	1.000	–	1.000
Married		279	114 (40.9%)	<0.001	3.455 (1.819–6.562)	0.026	2.206 (1.100–4.425)
Divorced/widowed		159	60 (37.7%)	0.001	3.030 (1.541–5.960)	0.201	1.624 (0.773–3.414)
Education				<0.001		0.067	
Senior middle school and above		25	3 (12.0%)	–	1.000	–	1.000
Junior middle school		84	22 (26.2%)	0.150	2.602 (0.709–9.554)	0.220	2.429 (0.588–10.032)
Primary school		314	114 (36.3%)	0.022	4.180 (1.224–14.272)	0.123	2.926 (0.747–11.456)
Illiteracy		93	48 (51.6%)	0.002	7.822 (2.190–27.937)	0.031	4.681 (1.155–18.962)
Occupation							
Others		84	22 (26.2%)	–	1.000	–	1.000
Farming		432	165 (38.2%)	0.038	1.742(1.032–2.94)	0.038	1.742 (1.032–2.94)
Infection routes				0.929			
Heterosexual contact		507	186 (36.7%)	–	1.000		
Homosexual contact		7	0 (0.0%)	0.999	–		
Others		2	1 (50.0%)	0.700	1.726 (0.107–27.754)		

**Figure 3.**
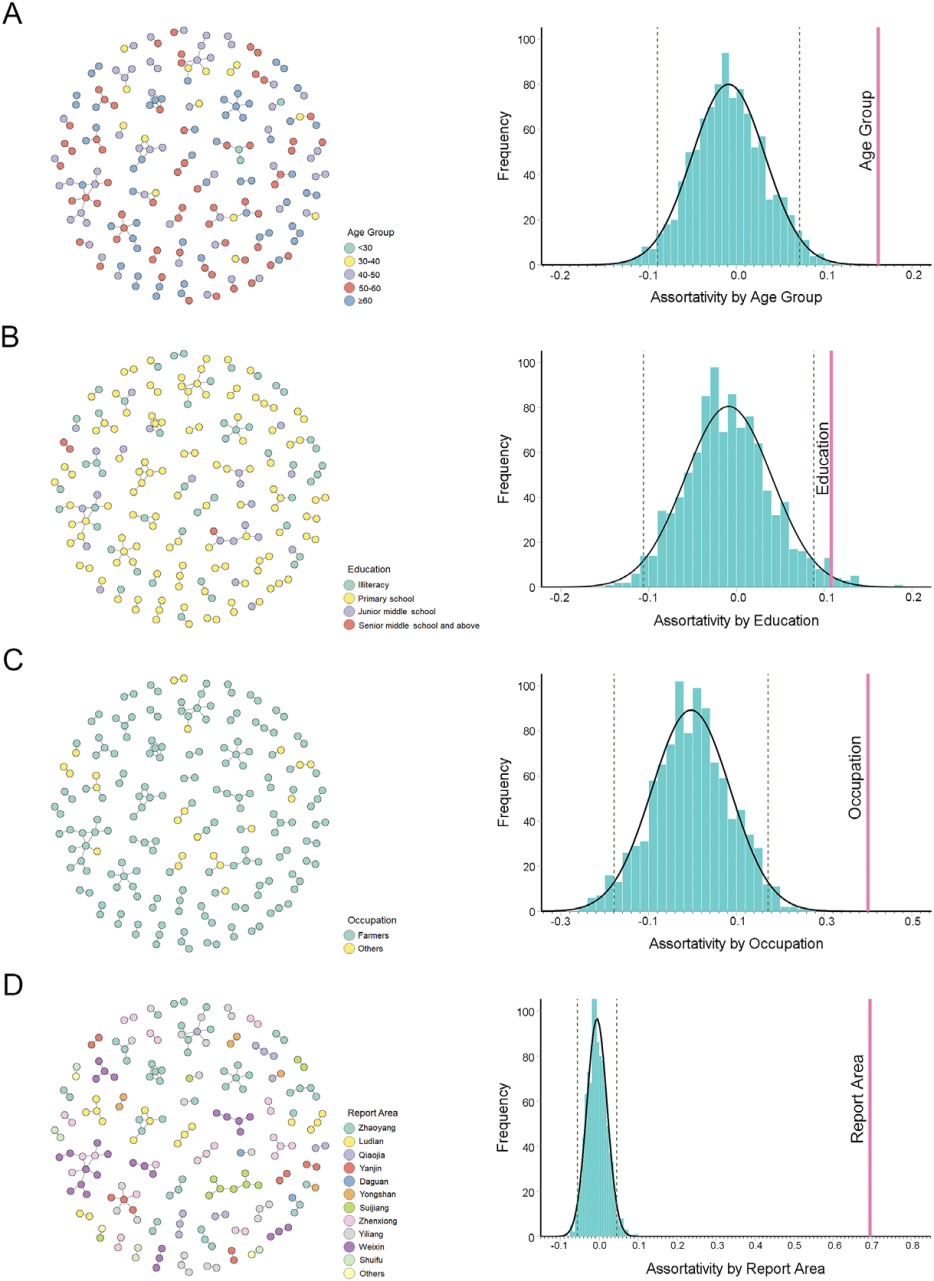
Assortativity analysis of the linked individuals in the HIV-1 molecular network in Zhaotong City. (a) Left panel: HIV-1 molecular clusters coded by age group. Right panel: assortativity analysed by age group. (b) Left panel: HIV-1 molecular clusters coded by educational level. Right panel: assortativity analysed by education level. (c) Left panel: HIV-1 molecular clusters coded by occupation. Right panel: assortativity analysed by occupation. (d) Left panel: HIV-1 molecular clusters coded by report area. Right panel: assortativity analysed by report area. The grey dotted line represents the 5% significance level. The pink vertical line represents the corresponding assortativity.

To further investigate the common features of the transmission clusters, the assortativity coefficient (*r_a_*) of the demographic characteristics was analysed (Figure 3). The results showed that individuals in the same age group (*r_a_* = 0.166, *p* < 0.05), with a similar educational level (*r_a_* = 0.108, *p* < 0.05), with the same occupation (*r_a_* = 0.400, *p* < 0.05), and reported in the same county (*r_a_* = 0.695, *p* < 0.05) were more likely to be genetically linked.

## Discussion

In this study, a cross-sectional HIV-1 molecular epidemiological survey was conducted in Zhaotong City, an area severely affected by HIV-1 in Yunnan Province, which revealed the demographic and spatial distributions of HIV-1 genotypes in the local area. HIV-1 molecular network analysis revealed the characteristics and risks of HIV-1 transmission.

The dominant HIV-1 strains circulating in China originate mainly in Yunnan Province [19]. CRF01_AE was first introduced into Yunnan from Thailand [28]. CRF07_BC and CRF08_BC are considered to be formed in Yunnan Province [29]. Similar to other parts of Yunnan Province [30], CRF08_BC, CRF07_BC, and CRF01_AE were also the predominant strains in Zhaotong City. According to a national HIV-1 molecular epidemiology survey, CRF07_BC was the most prevalent strain in China [31, 32]. In the neighbouring provinces of Sichuan and Guizhou, CRF07_BC was the predominant HIV-1 genotype [33, 34]. However, CRF08_BC was the most prevalent strain in Yunnan, which appears to be a feature of the HIV-1 epidemic in Yunnan Province. In this study, the demographic analysis showed that CRF08_BC was most likely to be found in individuals whose household registration address was Zhaotong City, illiterate, and those with occupations other than farming. Individuals with other occupations included workers, clerks, civil servants, students, and service workers, which widened the social range of HIV transmission. Meanwhile, CRF08_BC showed a tendency for spatial clustering, with a high–high cluster area detected in Zhaoyang County, the capital city of Zhaotong City, suggesting that CRF08_BC had the characteristics of an endemic epidemic. However, CRF07_BC did not exhibit significant demographic or spatial distribution characteristics.

Initially, CRF01_AE was found mainly in FSWs in Yunnan Province [35]. Therefore, it was thought to be transmitted through heterosexual contact. With the increase in CRF08_BC in heterosexual transmission, the proportion of CRF01_AE in heterosexual transmission remained low [30]. In this study, CRF01_AE was most likely found in married individuals, who may represent a specific subgroup of heterosexual transmission. With the increase in homosexual transmission, CRF01_AE has been reported to be one of the predominant strains in men who have sex with men [36]. This study also found that the proportion of CRF01_AE was higher in homosexual transmissions. In terms of spatial distribution, the cluster of CRF01_AE was detected in the eastern region of Zhaotong City, including Zhenxiong and Weixin counties. These two counties border Luzhou City in Sichuan Province, where CRF01_AE is the predominant strain [33]. This suggests that interregional transmission may occur.

Owing to a prolonged epidemic, HIV-1 genetic recombination is a universal phenomenon in Yunnan Province, including the high frequency of URFs and the emergence of novel CRFs, which have contributed to the diversity of HIV-1 genetics. In this study, the proportion of URFs ranked fourth. Although BC recombination was dominant, some second-generation recombination also occurred from the dominant CRFs. Among the six novel CRFs detected in this study, four (CRF64_BC, CRF101_ 01 B, CRF118_BC, and CRF125_0107) were identified for the first time in Yunnan Province. One of the three near-full-length sequences used for the nomenclature of CRF101_ 01 B originated from Zhaotong City [37]. CRF125_0107 was recently identified among heterosexuals in Zhaotong City [38]. This study confirms the local transmission of these two CRFs. The other two (CRF55_01B and CRF85_BC) were first detected in other provinces. CRF55_01B was considered the first CRFs circulating mainly among MSM [39], but is now found in the heterosexual contact population. CRF85_BC was first identified among heterosexuals in Yibin City, Sichuan Province, and remains the most prevalent strain in Yibin [33, 40]. Although the global spatial autocorrelation analysis of CRF85_BC showed no statistical difference, it appeared that CRF85_BC was mainly distributed in the three counties (Shuifu, Yanjin, and Weixin) bordering Yibin, suggesting cross-regional transmission of this CRF.

In this study, a basic molecular network was constructed with newly reported HIV cases. Compared with individuals from other provinces, Zhaotong natives were more likely to be found in the network, suggesting that the network was localised and that the epidemic was endemic. Compared to unmarried individuals, married individuals were more likely to be found in the network. The proportion of commercial heterosexual contacts was higher among unmarried individuals (42.3%, 33/78) than among married individuals (30.5%, 85/279). Meanwhile, 11.5% of the married respondents had marital transmission. The data showed that the proportion of individuals detected in networks with commercial heterosexual contact (30.7%, 51/166) was lower than the proportion of those with non-marital non-commercial heterosexual contact (39.2%, 121/309) and those transmitting between spouses (43.8%, 14/32), probably because commercial heterosexual behaviour was relatively covert and infection tracing was not easy to trace. This may explain why married people are more likely to be found in networks, but further investigation is needed. In addition, spousal notification and positive prevention measures need to be strengthened.

Zhaotong City is an underdeveloped area in Yunnan Province, and the education level of its population is low. Low literacy leads to low awareness of HIV/AIDS [41]. Among the participants, 78.9% had a primary school education or lower. As the level of education decreased, the proportion of people in networks increased, especially among illiterates. In recent years, the age of people newly diagnosed with HIV infection in China has gradually increased. In this study, more than half of the reported HIV-infected individuals were older than 50 years. In addition, people aged ≥60 years were more likely to be detected in networks than in other age groups. This suggests that older people are a large high-risk group in the transmission network. In fact, the proportion of illiterate people increased with age (trend *χ* ^2^ = 24.064, *p* < 0.001) and was the highest among those aged 60 years and older (29.6%, 29/98). A low level of education leads to a lack of knowledge about AIDS in older age groups, which increases the incidence of HIV-related high-risk sexual behaviour [42]. Meanwhile, high-risk behaviours in this subpopulation were as complex as in any other age group, including commercial heterosexual contact (36.7%, 36/98), non-commercial heterosexual contact (54.1%, 53/98), and transmission between spouses or fixed sexual partners (7.1%, 7/98). Therefore, targeted intervention and counselling services for older adults should be developed and made available in the community.

As the methodology evolves, molecular networks are increasingly combined with social networks to estimate the transmission characteristics [43, 44]. Some statistical methods from social network analysis have also been applied to molecular network analysis, including individual- and network-level metrics [45]. In this study, a network-level indicator, assortativity, was attempted. Assortativity assesses whether nodes with the same attributes tend to connect with one another. If assortativity is positive, people with the same characteristics tend to come together in social networks. In this study, the assortativity of reporting areas was higher, suggesting that HIV-1 cases from each county tended to cluster separately. Therefore, it is necessary to develop preventive strategies and policies based on the characteristics of different countries. Meanwhile, cases with the same age, education level, and occupation tended to cluster separately and formed different subpopulations. Thus, it may be more effective to implement interventions according to the characteristics of subpopulations, such as developing core prevention and control knowledge points for subpopulations and implementing peer education and counselling.

In the context of a long-term HIV-1 epidemic, this study used a cross-sectional HIV-1 molecular epidemiological study to reveal the complicated epidemic characteristics of HIV-1 in Zhaotong City. Multiple HIV-1 strains co-circulate in the local area, among which CRF01_AE and CRF08_BC show a unique population and spatial distribution. In particular, HIV-1 molecular network analysis provides an opportunity for an in-depth analysis of transmission characteristics and potential risks by constructing individual transmission correlations. The analysis suggested that some subpopulations were at a specific risk and should be targeted, such as infections in the elderly, illiterate, and married. People with some of the same characteristics tended to be genetically linked, suggesting that holistic interventions may be effective. In short, this study provides clues and directions for further targeted detection and intervention.

## Supporting information

Cao et al. supplementary material Cao et al. supplementary material

## Data Availability

The datasets used and/or analysed in the current study are available upon request from the corresponding author.

## Supplementary material

For supplementary material accompanying this paper visit http://doi.org/10.1017/S0950268823001140. click here to view supplementary material
