# Supplementary material for: Using molecular network analysis to understand current HIV-1 transmission characteristics in an inland area of Yunnan, China
Source: Epidemiol Infect. 2023 Jul 18;151:e124. doi: 10.1017/S0950268823001140 (PMC10540185; doi:10.1017/S0950268823001140)
Supplement: Cao et al. supplementary material [file S0950268823001140sup001.pdf]

Supplementary Material

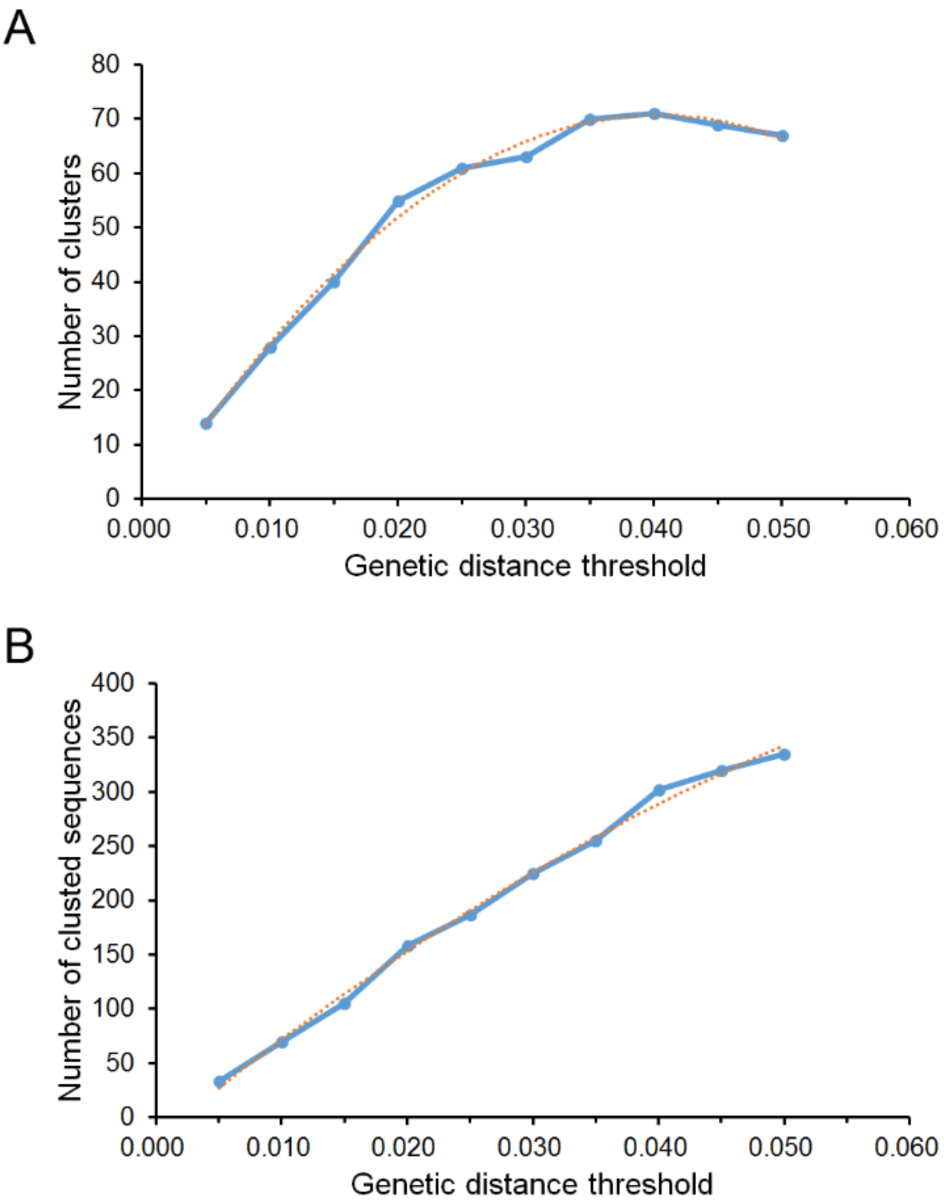

Supplementary Figure S1. Evaluation of the effect of the genetic distance threshold on cluster identification. A, Number of clusters changing with an increasing of genetic distance threshold. The dashed line is the trend line. B, Number of clustered sequences changing with an increase of genetic distance threshold. The dashed line is the trend line.

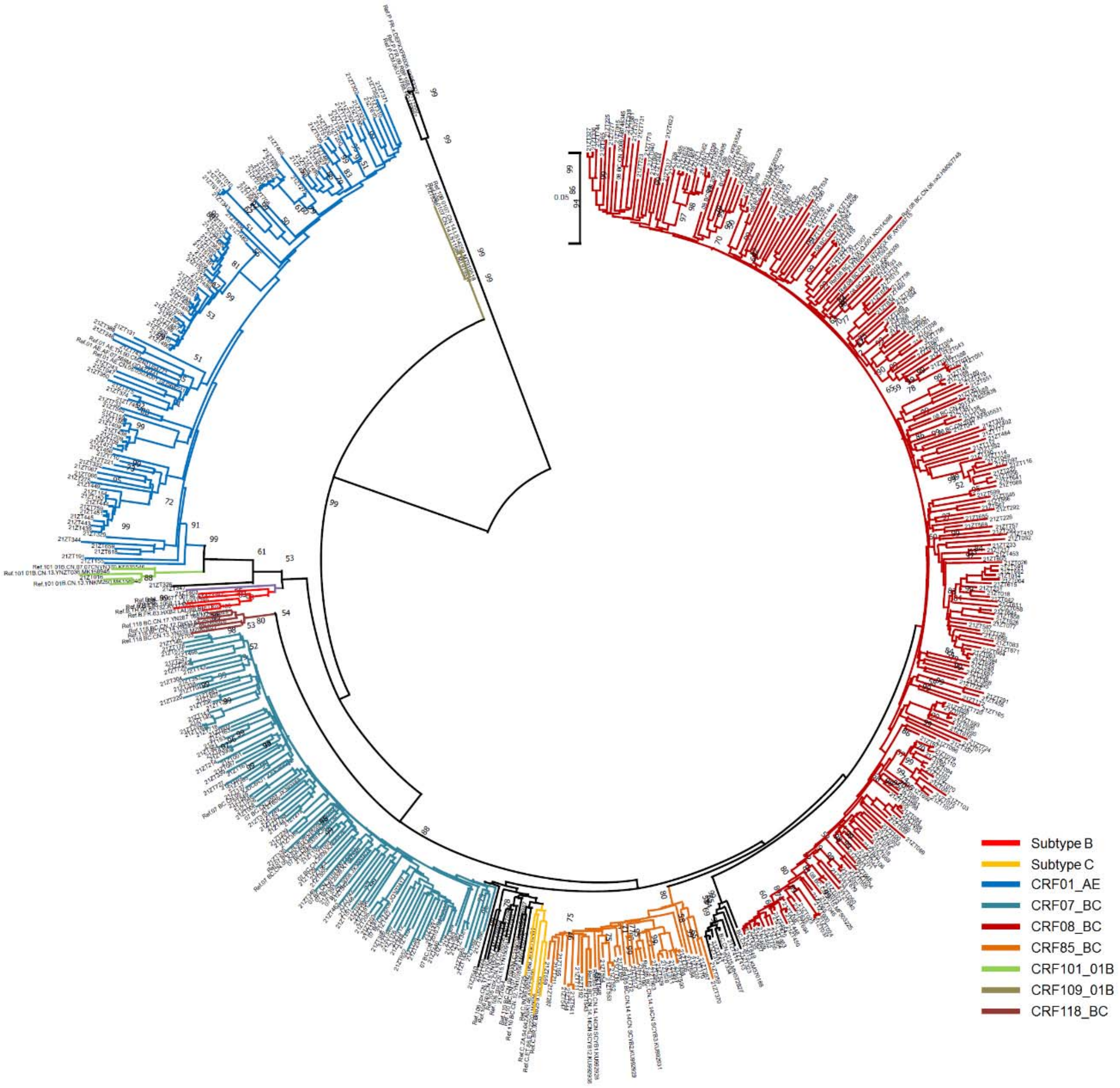

Supplementary Figure S2. Neighbor-joining phylogenetic tree of the partial *gag* gene. The scale bar indicates 5% nucleotide sequence divergence. Values on the branches represent the percentage of 1000 bootstrap replicates. The dataset for this analysis includes 495 sequences (accession number: OP979115-OP979609) from this study and 59 reference sequences from HIV Databases (http://hiv-web.lanl.gov/content/index). The different colors are used to label the different subtypes/CRFs. Except for the black branches, one color represents one subtype/CRF, including sequences from this study and reference sequences. The black branches include eight potentially recombinant sequences from this study and the reference sequences of CRF106_BC, CRF110_BC and Group P (Outgroup). Because the clades of CRF106_BC, CRF110_BC and Group P don’t include sequences from this study, they are not individually colored.

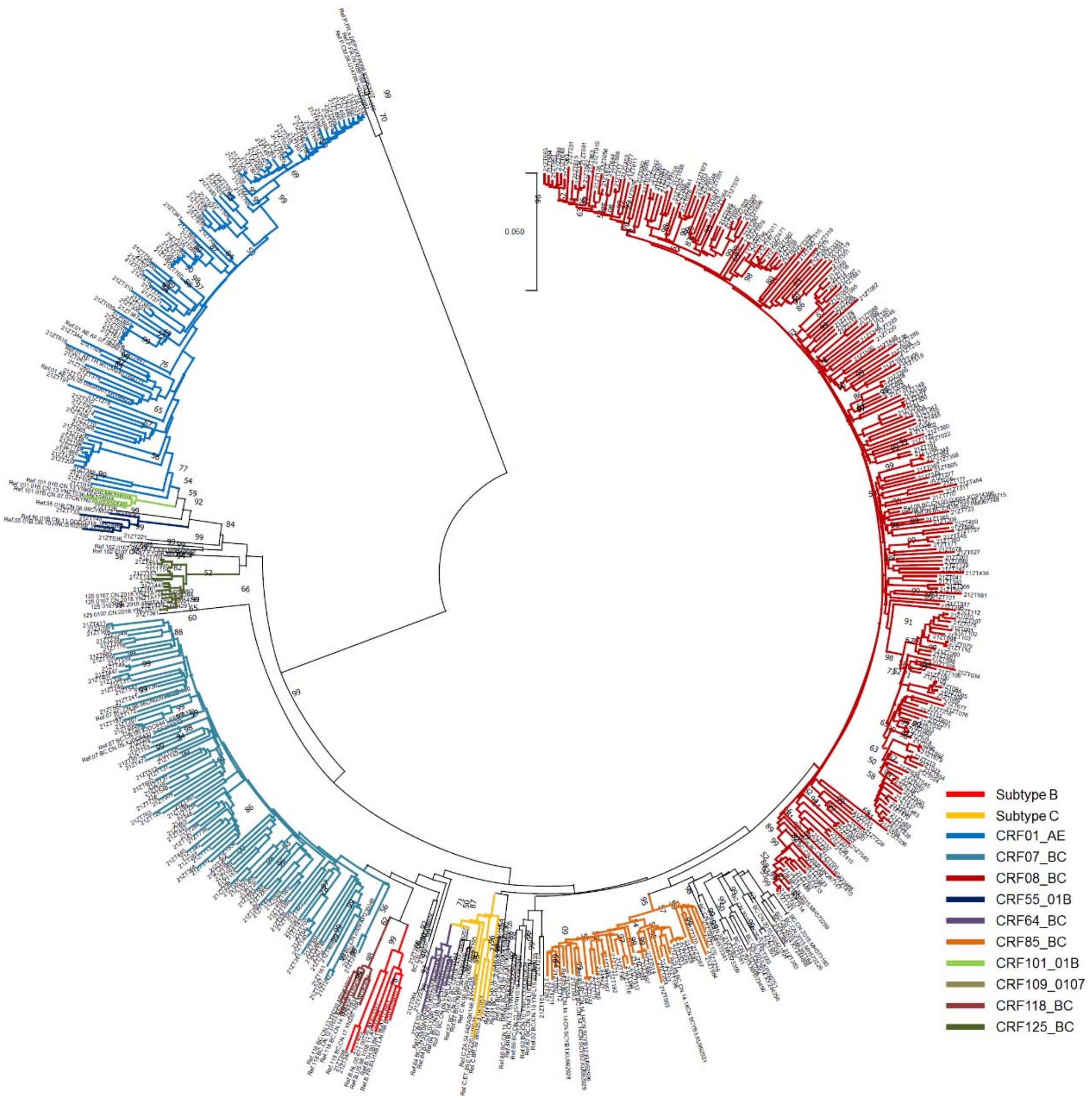

Supplementary Figure 3S. Neighbor-joining phylogenetic tree of the partial *pol* gene. The scale bar indicates 5% nucleotide sequence divergence. Values on the branches represent the percentage of 1000 bootstrap replicates. The dataset for this analysis includes 516 *pol* sequences (accession number: OP979610-OP980125) from this study and 74 reference sequences from HIV Databases (http://hiv-web.lanl.gov/content/index). The different colors are used to label the different subtypes/CRFs. Except for the black branches, one color represents one subtype/CRF, including sequences from this study and reference sequences. The black branches include nine potentially recombinant sequences from this study and the reference sequences of CRF57_BC, CRF61_BC, CRF62_BC, CRF86_BC and Group P (Outgroup). Because the clades of CRF57_BC, CRF61_BC, CRF62_BC, CRF86_BC and Group P don't include sequences from this study, they are not individually colored.

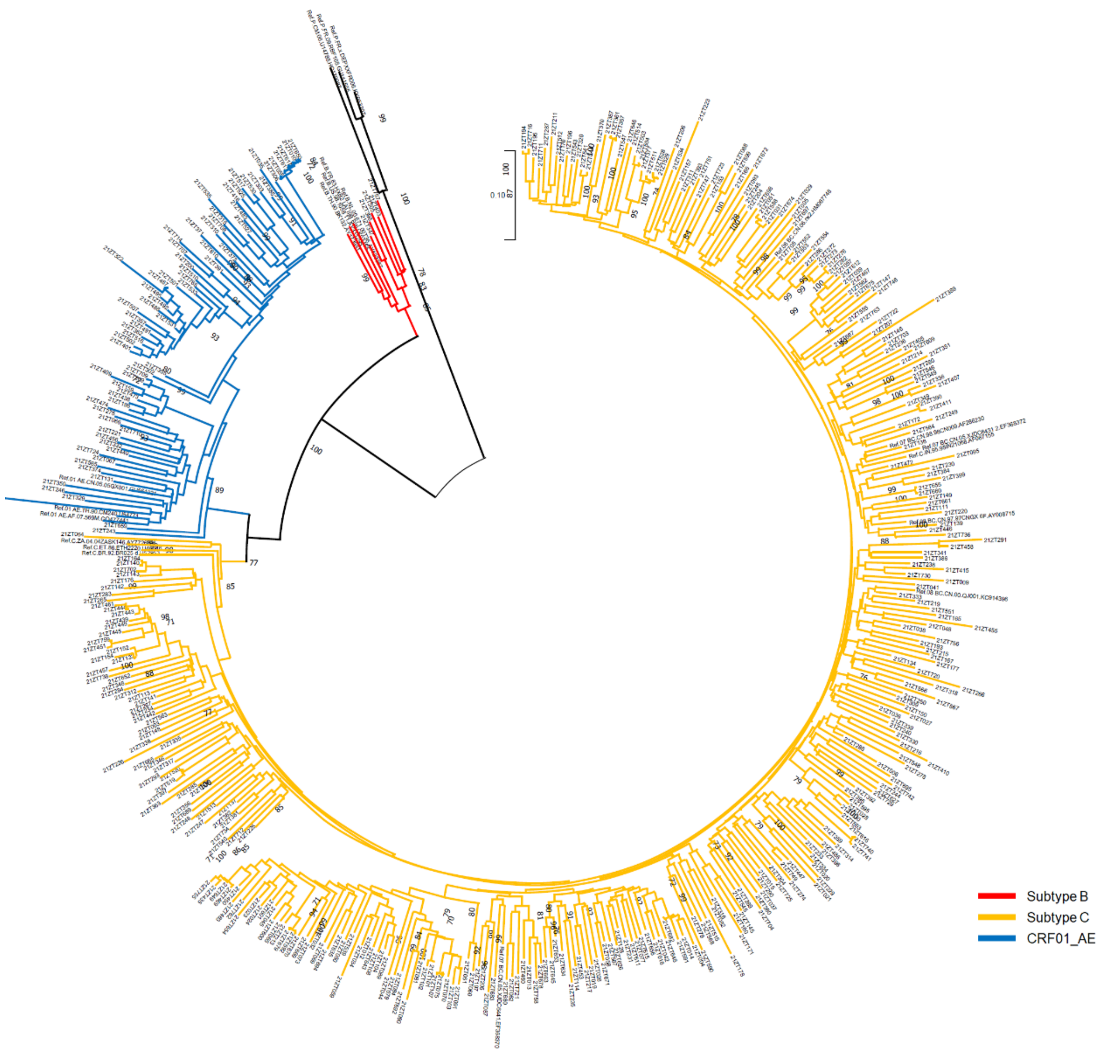

Supplementary Figure S4. Neighbor-joining phylogenetic tree of the partial *env* gene. The scale bar indicates 10% nucleotide sequence divergence. Values on the branches represent the percentage of 1000 bootstrap replicates. The dataset for this analysis includes 414 *env* sequences (accession number: OP980126-OP980539) from this study and 20 reference sequences from HIV Databases (http://hiv-web.lanl.gov/content/index). The different colors are used to label the different subtypes/CRFs. Except for the black branches, one color represents one subtype/CRF, including sequences from this study and reference sequences. The black branches represent the reference sequences of Group P (Outgroup).

Supplementary Table S1. The constituent of genetic region combinations.

Genetic region combinations	Number
gag+pol+env	292
gag+pol	141
gag+env	46
pol+env	63
Total	542

Supplementary Table S2. The comparison of demographic characteristics of subjects with subtypes and without subtypes.

Characteristics	Subjects with subtypes	Subjects without subtypes	χ2	*P*
**Total**	542	121		
**Report area**			17.009	0.108
Zhaoyang	141 (75.4%)	46 (24.6%)		
Ludian	41 (83.7%)	8 (16.3%)		
Qiaojia	42 (87.5%)	6 (12.5%)		
Yanjin	25 (89.3%)	3 (10.7%)		
Daguan	17 (85.0%)	3 (15.0%)		
Yongshan	30 (85.7%)	5 (14.3%)		
Suijiang	26 (89.7%)	3 (10.3%)		
Zhenxiong	97 (77.6%)	28 (22.4%)		
Yiliang	44 (81.5%)	10 (18.5%)		
Weixin	52 (92.9%)	4 (7.1%)		
Shuifu	16 (88.9%)	2 (11.1%)		
Others	11 (78.6%)	3 (21.4%)		
**Registered residence**			1.290	0.525
Zhaotong	497 (82.1%)	108 (17.9%)		
Other cities in Yunnan	7 (87.5%)	1 (12.5%)		
Other provinces	38 (76.0%)	12 (24.0%)		
**Gender**			0.009	0.925
Male	392 (81.8%)	87 (18.2%)		
Female	150 (81.5%)	34 (18.5%)		
**Age**			3.905	0.419
<30	30 (71.4%)	12 (28.6%)		
30-39	85 (80.2%)	21 (19.8%)		
40-49	149 (82.3%)	32 (17.7%)		
50-59	173 (84.0%)	33 (16.0%)		
≥60	105 (82.0%)	23 (18.0%)		
**Race/ethnicity**			0.079	0.778
Han	497 (81.9%)	110 (18.1%)		
Others	45 (80.4%)	11 (19.6%)		
**Marital Status**			3.425	0.180
Unmarried	82 (82.0%)	18 (18.0%)		
Married	286 (79.4%)	74 (20.6%)		
Divorced/Widowed	174 (85.7%)	29 (14.3%)		
**Education**			3.945	0.267
Illiteracy	100 (77.5%)	29 (22.5%)		
Primary school	327 (83.4%)	65 (16.6%)		
Junior middle school	90 (78.9%)	24 (21.1%)		
Senior middle school and above	25 (89.3%)	3 (10.7%)		

**Occupation**			0.925	0.336
Farmers	450 (82.4%)	96 (17.6%)		
Others	92 (78.6%)	25 (21.4%)		
**Infection Routes**			1.155	0.570
Heterosexual contact	533 (81.5%)	121 (18.5%)		
Homosexual contact	7 (100.0%)	0 (0.0%)		
Unknown	2 (100.0%)	0 (0.0%)		

Supplementary Table S3. Demographic characteristics associated with HIV-1 genotypes (univariate analysis).

Characteristics	Total	The number of specific genotype (%)	Univariate analysis	
			P	OR (95%CI)
**CRF01_AE**				
**Registered residence**			0.954	
Other cities in Yunnan	7	1 (14.3%)	-	1.000
Zhaotong	497	84 (16.9%)	0.855	1.220 (0.145-10.269)
Other provinces	38	7 (18.4%)	0.793	1.355 (0.140-13.118)
**Gender**				
Male	392	65 (16.6%)	-	1.000
Female	150	27 (18.0%)	0.694	1.104 (0.674-1.810)
**Age**			0.046	
≥60	105	19 (18.1%)	-	1.000
50-59	173	34 (19.7%)	0.749	1.107 (0.594-2.063)
40-49	149	19 (12.8%)	0.242	0.662 (0.331-1.321)
30-39	85	10 (11.8%)	0.231	0.604 (0.264-1.378)
<30	30	10 (33.3%)	0.078	2.263 (0.914-5.607)
**Race/ethnicity**				
Han	497	82 (16.5%)	-	1.000
Others	45	10 (22.2%)	0.330	1.446 (0.689-3.036)
**Marital Status**			0.042	
Divorced/Widowed	174	20 (11.5%)	-	1.000
Married	286	59 (20.6%)	0.013	2.001 (1.158-3.458)
Unmarried	82	13 (15.9%)	0.333	1.451 (0.683-3.083)
**Education**			0.105	
Illiteracy	100	11 (11.0%)	-	1.000
Primary school	327	54 (16.5%)	0.182	1.600 (0.802-3.194)
Junior middle school	90	22 (24.4%)	0.017	2.618 (1.188-5.765)
Senior middle school and above	25	5 (20.0%)	0.235	2.023 (0.632-6.472)
**Occupation**				
Others	92	15 (16.3%)	-	1.000
Farmers	450	77 (17.1%)	0.851	1.060 (0.579-1.941)
**Infection Routes**			0.024	
Heterosexual contact	533	87 (16.3%)	-	1.000
Homosexual contact	7	4 (57.1%)	0.013	6.835 (1.503-31.080)
Others	2	1 (50.0%)	0.249	5.126 (0.318-82.741)
**CRF07_BC**				
**Registered residence**			0.018	
Zhaotong	497	78 (15.7%)	-	1.000
Other cities in Yunnan	7	1 (14.3%)	0.919	0.895 (0.106-7.540)
Other provinces	38	13 (34.2%)	0.005	2.793 (1.370-5.696)

**Gender**				
Female	150	22 (14.7%)	-	1.000
Male	392	70 (17.9%)	0.377	1.265 (0.751-2.13)
**Age**			0.001	
≥60	105	11 (10.5%)	-	1.000
50-59	173	22 (12.7%)	0.576	1.245 (0.578-2.684)
40-49	149	25 (16.8%)	0.160	1.723 (0.807-3.677)
30-39	85	23 (27.1%)	0.004	3.170 (1.443-6.963)
<30	30	11 (36.7%)	0.001	4.947 (1.875-13.055)
**Race/ethnicity**				
Others	45	5 (11.1%)	-	1.000
Han	497	87 (17.5%)	0.279	1.698 (0.651-4.425)
**Marital Status**			0.001	
Married	286	36 (12.6%)	-	1.000
Divorced/Widowed	174	31 (17.8%)	0.125	1.505 (0.893-2.538)
Unmarried	82	25 (30.5%)	<0.001	3.046 (1.696-5.471)
**Education**			0.024	
Illiteracy	100	8 (8.0%)	-	1.000
Primary school	327	59 (18.0%)	0.019	2.532 (1.166-5.498)
Junior middle school	90	17 (18.9%)	0.031	2.678 (1.095-6.552)
Senior middle school and above	25	8 (32.0%)	0.003	5.412 (1.787-16.392)
**Occupation**				
Others	92	15 (16.3%)	-	1.000
Farmers	450	77 (17.1%)	0.851	0.944 (0.515-1.728)
**Infection Routes**			0.120	
Heterosexual contact	533	88 (16.5%)	-	1.000
Homosexual contact	7	3 (42.9%)	0.084	3.793 (0.834-17.242)
Others	2	1 (50.0%)	0.253	5.057 (0.313-81.611)
**CRF08_BC**				
**Registered residence**			0.033	
Other provinces	38	10 (26.3%)	-	1.000
Zhaotong	497	241 (48.5%)	0.011	2.636 (1.254-5.542)
Other cities in Yunnan	7	4 (57.1%)	0.120	3.733 (0.708-19.674)
**Gender**				
Male	392	180 (45.9%)	-	1.000
Female	150	75 (50.0%)	0.395	1.178 (0.808-1.717)
**Age**			0.037	
<30	30	6 (20.0%)	-	1.000
30-39	85	36 (42.4%)	0.033	2.939 (1.089-7.930)
40-49	149	74 (49.7%)	0.005	3.947 (1.526-10.210)
50-59	173	89 (51.4%)	0.003	4.238 (1.651-10.882)
≥60	105	50 (47.6%)	0.009	3.636 (1.374-9.623)
**Race/ethnicity**				
Han	497	230 (46.3%)	-	1.000

Others	45	25 (55.6%)	0.235	1.451 (0.785-2.681)
**Marital Status**			0.066	
Unmarried	82	29 (35.4%)	-	1.000
Married	286	138 (48.3%)	0.040	1.704 (1.025-2.834)
Divorced/Widowed	174	88 (50.6%)	0.023	1.870 (1.088-3.214)
**Education**			0.004	
Senior middle school and above	25	9 (36.0%)	-	1.000
Junior middle school	90	36 (40.0%)	0.717	1.185 (0.473-2.971)
Primary school	327	147 (45.0%)	0.387	1.452 (0.624-3.381)
Illiteracy	100	63 (63.0%)	0.017	3.027 (1.216-7.535)
**Occupation**				
Farmers	450	202 (44.9%)	-	1.000
Others	92	53 (57.6%)	0.027	1.668 (1.060-2.625)
**Infection Routes**			1.000	
Heterosexual contact	533	255 (47.8%)	-	1.000
Homosexual contact	7	0 (0.0%)	0.999	-
Others	2	0 (0.0%)	0.999	-
**CRF85_BC**				
**Registered residence**			0.941	
Zhaotong	497	32 (6.4%)	-	1.000
Other provinces	38	3 (7.9%)	0.727	1.246 (0.363-4.271)
Other cities in Yunnan	7	0 (0.0%)	0.999	-
**Gender**				
Male	392	24 (6.1%)	-	1.000
Female	150	11 (7.3%)	0.608	1.213 (0.579-2.543)
**Age**			0.574	
30-39	85	5 (5.9%)	-	1.000
40-49	149	12 (8.1%)	0.540	1.401 (0.476-4.123)
50-59	173	8 (4.6%)	0.665	0.776 (0.246-2.447)
>=60	105	10 (9.5%)	0.359	1.684 (0.553-5.131)
<30	30	0 (0.0%)	0.998	-
**Race/ethnicity**				
Others	45	1 (2.2%)	-	1.000
Han	497	34 (6.8%)	0.253	3.231 (0.432-24.174)
**Marital Status**			0.172	
Unmarried	82	1 (1.2%)	-	1.000
Married	286	20 (7.0%)	0.080	6.090 (0.805-46.081)
Divorced/Widowed	174	14 (8.0%)	0.061	7.087 (0.916-54.851)
**Education**			0.374	
Illiteracy	100	5 (5.0%)	-	1.000
Primary school	327	26 (8.0%)	0.324	1.641 (0.613-4.393)
Junior middle school	90	3 (3.3%)	0.570	0.655 (0.152-2.823)
Senior middle school and above	25	1 (4.0%)	0.835	0.792 (0.088-7.097)
**Occupation**				

Others	92	2 (2.2%)	-	1.000
Farmers	450	33 (7.3%)	0.085	3.561 (0.839-15.111)
**Infection Routes**			1.000	
Heterosexual contact	533	35 (6.6%)	-	1.000
Homosexual contact	7	0 (0.0%)	0.999	-
Others	2	0 (0.0%)	0.999	-
**URFs**				
**Registered residence**			0.861	
Other provinces	38	3 (7.9%)	-	1.000
Zhaotong	497	43 (8.7%)	0.873	1.105 (0.326-3.742)
Other cities in Yunnan	7	1 (14.3%)	0.591	1.944 (0.172-21.937)
**Gender**				
Female	150	10 (6.7%)	-	1.000
Male	392	37 (9.4%)	0.307	1.459 (0.706-3.014)
**Age**			0.361	
<30	30	1 (3.3%)	-	1.000
30-39	85	11 (12.9%)	0.171	4.311 (0.532-34.911)
40-49	149	15 (10.1%)	0.263	3.246 (0.412-25.563)
50-59	173	11 (6.4%)	0.524	1.969 (0.245-15.840)
>=60	105	9 (8.6%)	0.352	2.719 (0.331-22.365)
**Race/ethnicity**				
Han	497	43 (8.7%)	-	1.000
Others	45	4 (8.9%)	0.957	1.030 (0.352-3.013)
**Marital Status**			0.235	
Married	286	21 (7.3%)	-	1.000
Divorced/Widowed	174	15 (8.6%)	0.621	1.190 (0.596-2.376)
Unmarried	82	11 (13.4%)	0.090	1.955 (0.901-4.244)
**Education**			0.537	
Senior middle school and above	25	1 (4.0%)	-	1.000
Junior middle school	90	7 (7.8%)	0.519	2.024 (0.237-17.272)
Primary school	327	27 (8.3%)	0.459	2.160 (0.281-16.591)
Illiteracy	100	12 (12.0%)	0.266	3.273 (0.405-26.442)
**Occupation**				
Others	92	5 (5.4%)	-	1.000
Farmers	450	42 (9.3%)	0.232	1.791 (0.689-4.658)
**Infection Routes**				
Heterosexual contact	533	47 (8.8%)	-	1.000
Homosexual contact	7	0 (0.0%)	0.999	-
Others	2	0 (0.0%)	0.999	-
